# Comprehensive analysis of college students' autonomous fitness behavior—a narrative review

**DOI:** 10.3389/fspor.2024.1406810

**Published:** 2024-05-21

**Authors:** Zhendong Gao, Chen Soon Chee, Roxana Dev Omar Dev, Jianhong Gao

**Affiliations:** Department of Sports Studies, Faculty of Educational Studies, Universiti Putra Malaysia, Serdang, Malaysia

**Keywords:** autonomous fitness behavior, college students, Self-Determination Theory, physical health, agency

## Abstract

Although the physical health of college students is increasingly receiving attention, their autonomous fitness behavior has not been thoroughly investigated. This narrative review conducted a comprehensive literature search through databases such as PubMed, PsycINFO, Web of Science, and the China National Knowledge Infrastructure (CNKI), reviewing studies published up to December 2023. We explored the constructs of autonomy, fitness behavior, and agency, and discussed their integration within the autonomous fitness model. Our findings indicate a lack of comprehensive studies exploring the multifaceted factors influencing autonomous fitness behaviors. Future research should strive to deepen conceptual understanding and further explore the complex dynamics of the transition from autonomy to persistence, employing technological and interdisciplinary methodological perspectives to enhance understanding and promote sustainable fitness habits.

## 1 Introduction

With the rise of global health consciousness, individual health, particularly the physical exercise and fitness of young people, has become a focal point of public health research (1–3). As the future backbone of society, the health status of college students has garnered extensive attention (4–6). Their health behaviors not only impact their current study and life but also have a profound influence on their future health (7–9). However, it appears that the issue of insufficient physical activity and declining physical health among college students has not yet been fully addressed (10, 11).

In recent years, with the advancement of technology and the widespread dissemination of health education (12–14), an increasing number of college students have begun to spontaneously engage in fitness activities, including working out alone, participating in group sports, and using fitness applications (5, 15, 16). From the perspective of agency, the stages of a person's fitness behavior can be divided into four phases: the transition from passive to active, then to autonomous, and finally to automatic (passive-active-autonomous-automatic) (17). Given the potential benefits of continuous physical activity participation in enhancing physical health, psychological well-being, and social skills (18), promoting sustained or regular physical exercise among college students is considered a focus for future research (10, 19). Autonomy, as a precursor to automation, appears to provide a causal mechanism for understanding the sustained or long-term participation in physical activities by college students. However, most literature reviews have concentrated on college students' physical activities, exercise behaviors, or physical health. There has been a lack of research focusing on the literature review of college students' autonomous fitness behaviors. Therefore, this narrative review will provide a clear outline for future research on college students' autonomous fitness behaviors based on existing literature, through conceptual clarification and review of research progress, point out potential research gaps, and offer theoretical guidance for practice.

## 2 Methods

A comprehensive literature search was conducted up to December 2023 using PubMed, PsycINFO, Web of Science, and the China National Knowledge Infrastructure (CNKI). Keywords such as “autonomous fitness behavior” were combined using Boolean operators to expand the search scope. This review included studies in both English and Chinese to capture diverse perspectives and developments in the field.

Studies were selected based on their focus on autonomous fitness behaviors in college students, particularly those discussing or utilizing Self-Determination Theory or related constructs. Given the exploratory nature of this narrative review, the selection of articles was primarily guided by their relevance to the central themes of autonomy and fitness behavior among college students. While this approach allowed for a broad exploration of the topic, it was not constrained by strict inclusion or exclusion criteria typically used in systematic reviews (20).

Relevant information was extracted from the selected studies, including study objectives, design, theoretical framework, sample characteristics, and key findings. This data was synthesized to provide an overview of the conceptual and empirical landscape of autonomous fitness behaviors among college students, highlighting key theoretical insights and research advancements.

## 3 Conceptual clarification of autonomous fitness behavior

The concept of autonomous fitness behavior was first proposed and summarized by Chinese scholar Fang Rui, based on the theory of positive development in adolescents, aiming to address the lack of autonomy in the fitness behavior of youths under the backdrop of Chinese collectivism (21). Fang (17) employed qualitative research methods such as interpretative phenomenological analysis interviews, open-ended questionnaires, and dual hermeneutics, integrating concepts from Self-Determination Theory (SDT) (22) and Intentionality Self-Regulation (SOC theory) (23). She concluded that autonomous fitness behavior in adolescents is a form of exercise that is self-determined by the individual (acting subject), supported by the autonomy of the behavioral environment, and involves intentional self-regulation (i.e., consciously selecting, optimizing, and compensating for behavioral goals and means (SOC strategies) (17). This research has provided a solid theoretical framework and practical integration experience for subsequent studies.

In her research (17), the autonomous fitness behavior emerged from identifying the issue of passive participation in adolescent physical exercises. She then utilized the principles and methods of Interpretative Phenomenological Analysis (IPA) and the “sensitizing concepts” of symbolic interactionism to address these issues. This led to the development of a conceptual model of autonomous fitness behavior for adolescents, incorporating an agency perspective and integrating intentional self-regulation SOC strategies (17). The entire scientific research process and thought pattern are also worthy of study and emulation by researchers.

Autonomous fitness behavior is considered to be a person's conscious, voluntary, proactive, and self-controlled fitness behavior (17). It is based on the theory of self-determination, demonstrating the self-esteem and self-regulation capabilities of the individual engaged in fitness (24). Sheng et al. (25) views autonomous fitness behavior in adolescents as a descriptive concept that has evolved from the general notion of physical exercise behavior. It encompasses not only external and internal stimuli such as social roles, institutions, culture, behavioral norms, social situations, as well as individuals' sensations, motivations, and attitudes but also focuses on the subjective domain of the behavioral subject (25, 26). This includes paying attention to the active role of consciousness in behavior and the understanding and use of behavioral strategies by individuals during the process of behavior. The following study will interpret autonomy, fitness behavior, and the agency perspective to better understand the sensitized concept of autonomous fitness behavior.

### 3.1 Autonomy

As a precursor step to automatization, autonomy offers us a perspective on how to promote the development of sustained physical activity and exercise habits among college students. It's a concept worthy of further analysis and summary. Primarily, autonomy is a core concept in autonomous fitness behavior. According to Self-Determination Theory, autonomy refers to the individual's ability to make free choices and act according to their own will, unrestricted by external forces (27). In other words, autonomy is about the degree to which a person feels they can control their own actions and decisions, embodying the will and sense of self-determination to choose and carry out actions that align with personal interests and values. Behavioral patterns of autonomy contribute to increasing an individual's interest and motivation, reinforcing belief (28, 29).

In autonomous fitness behaviors, Fang (24) adopted the concept of autonomy from Self-Determination Theory, including identified, integrated, and intrinsic aspects. That is to say, autonomous behavior is the pursuit of self-determined actions, a form of behavior driven by autonomous motivation, fueled by intrinsic motivation, and internalized motivations (identified and integrated motivations) (27). Specifically, identified motivation refers to an individual participating in an activity because they recognize its value and importance, even if it is not personally enjoyable; integrated motivation occurs when the activity is fully aligned with the individual's values and self-identity, becoming an integral part of their life and self-concept; intrinsic motivation is when the individual engages in the activity purely for the enjoyment and satisfaction derived from the activity itself, independent of external rewards (27). The sense of self-determination in autonomous fitness behavior is the pursuit of satisfying three basic psychological needs, serving as the intrinsic driving mechanism for engaging in autonomous fitness behaviors (24).

However, it is important to note that in constructing the conceptual model of autonomous fitness behavior, Fang (24) equates the concept of competence in Self-Determination Theory (SDT) (30) with Bandura's (31) self-efficacy, referring to an individual's belief that they can perform a particular action or behavior at a certain level, confident in their ability to be competent in that behavior. Perceived competence and self-efficacy are often interchangeably used (32); however, research indicates that there are conceptual and statistical differences between the two. Perceived competence is conceived within the theoretical framework of Self-Determination Theory, while self-efficacy is rooted in the framework of Social Cognitive Theory (32). Some studies suggest that perceived competence seems to capture a dimension of self-efficacy in the context of physical activity because it relates specifically to personal capability assessments, but lacks the dimension of choosing physical activity despite obstacles (33).

Additionally, Ryan and Deci (30) argued that autonomy support is fundamental for individuals to transform and integrate external values into their own, or in other words, autonomy support is a key variable in promoting the internalization of motivation. The motivational mechanism of autonomy also includes the perception of autonomy support from the environment. Fang (24) categorizes this into perceptions of institutional and facilities support, interpersonal support, atmospheric support, and situational support. This approach focuses more on the broad environmental and personal factors that either support or inhibit autonomous behavior, making her methodology more comprehensive yet less specific. In contrast, the multidimensional concept of autonomy support could be referenced to employ a structured, detailed method for optimizing the measurement and implementation of autonomy support in fitness environments (34). This might be more directly applicable to structured settings where specific interventions can be planned and measured.

Overall, in the autonomous fitness behavior of college students, autonomy is crucial as it is closely related to the sustainability, effectiveness, and overall well-being of an individual's fitness endeavor (35–37).

### 3.2 Fitness behavior

The term “fitness” is considered to have a broad semantic field. On one hand, it refers to physical exercises performed to obtain or maintain a good physical form and composition (i.e., the process); on the other hand, it refers to a state of good vigor and physical health (i.e., the result) (38). In many instances, fitness behavior and exercise behavior are used interchangeably, but in certain contexts, they have subtle differences. Fitness behavior generally refers to activities aimed at improving or maintaining an individual's overall health and physical condition. It includes not only physical activities but also, in a broader sense, diet, rest, reducing sedentary behavior, and other behaviors that maintain a healthy lifestyle (39). Exercise behavior, on the other hand, typically refers to organized, planned, and repetitive physical activities designed to improve or maintain one or more components of physical fitness (such as cardiorespiratory endurance, muscle strength, flexibility, etc.) (40, 41). Thus, exercise behavior can be understood as a narrower process within the broader concept of fitness behavior. Moreover, as fitness includes both health-related physical fitness and performance-related physical fitness, the goals of fitness behavior should be more comprehensive, encompassing overall health and well-being, while the goals of exercise behavior are usually more focused on enhancing specific physical abilities or achieving specific physical health targets (40).

In Fang's (17) study, fitness behavior is considered to involve various sports practice activities that individuals engage in to meet their needs for enhanced physical fitness, promotion of mental and physical health, and social adaptation. These activities include various sports learning behaviors, recreational sports behaviors, physical exercise behaviors, and sports competition behaviors. Specifically, as a type of physical fitness activity with certain intensity, fitness behavior is carried out for health, leisure, social interaction, and other purposes (26). It can be understood as an interpretation and expansion of the narrow definition of fitness behavior. Zhang and Huang (42) emphasize that in the Chinese context, sports fitness is regarded as primarily physical practice, focusing on developing people's hobbies and specialties in sports fitness through the selection and learning of fitness and sports activities. It's about acquiring scientific methods of fitness, cultivating a civilized and healthy lifestyle, and developing the adaptability to persist in sports fitness in various environments. This interpretation also emphasizes the attribute nature of the term fitness. At the same time, they highlight that although there are slight differences in concepts such as sports activities, physical exercises, and fitness activities, the connotations are largely similar (42). This suggests that in the context of Chinese culture, fitness behavior can be narrowly interpreted without affecting the descriptive content of its connotations, aligning with the general academic consensus (40, 41, 43). However, the limitations of narrow interpretations still need to be considered.

In summary, when discussing the autonomous fitness behavior of college students, understanding the differences between fitness and exercise helps to more comprehensively understand their health and activity habits, as well as the various aspects that need to be considered when designing strategies to promote healthy behaviors.

### 3.3 Agency perspective

Social Cognitive Theory emphasizes viewing human development, adaptation, and change from the perspective of the individual agent, rejecting the dichotomy between human agency and social structure (44, 45). Bandura (46) understands the development process of individual agency as evolving from perceiving causal relationships between environmental events, to understanding causal relationships through action, and finally recognizing oneself as an agent of action. This development process underscores the importance of self-construction by the subject. Individuals are considered to have the capacity for organization, proactivity, self-regulation, and self-reflection (46), and emphasize the individual's ability to contribute to and influence their living environment. Moreover, agency highlights four core attributes: intentionality, forethought, reactiveness, and self-reflectiveness. These core attributes demonstrate that human thought possesses generativity, creativity, proactivity, and reflectivity (46). Furthermore, Social Cognitive Theory emphasizes that personal attributes and the attributes of the environments individuals happen to encounter may affect the nature, scope, and intensity of their lives (31, 44). That is, the impact generated by personal agency is constructed jointly with individual attributes and the environment. In summary, agency is a unique aspect of humans, a capacity for self-direction and self-transformation (46).

Explaining autonomous fitness behavior from the perspective of agency means focusing on how individuals actively choose, control, and influence their fitness behavior. Agency emphasizes the individual's autonomous choices, coping methods, and control over their own actions. Fang (17), from this perspective of agency, first categorized the stages of adolescents’ fitness behavior and discovered the transition process from passive to active, then to autonomous, and finally to automatic (passive-active-autonomous-automatic), as well as its motivational mechanisms, thus proposing the sensitized concept of autonomous fitness behavior. The intentionality self-regulation process is considered a specific manifestation of individuals exercising subjective agency (47, 48). SOC strategies, as regulatory strategies, describe the process of individuals contributing to their future development (49). In short, intentionality self-regulation strategies (SOC strategies) address how to approach the fitness process from the perspective of agency.

In summary, the perspective of agency emphasizes that individuals are active constructors of health behaviors (such as autonomous fitness behavior). They do not passively respond to external stimuli but actively shape and influence their own health and well-being. In research and practice, starting from the perspective of agency can help us better understand and promote effective and lasting autonomous fitness behavior.

## 4 Research progress

Fang (17) utilized interpretative phenomenological analysis and the concept of sensitization in qualitative research to interpret adolescent autonomous fitness behavior and preliminarily examine its conceptual model, thus providing a starting point for the theoretical framework of subsequent related research. Studies on autonomous fitness behavior among college students subsequently ensued. Research has ranged from early assessments of autonomous fitness behavior in college students (50) to studies of the horizontal and longitudinal relationships of other significant variables, such as the relationship between exercise disincentives, exercise self-efficacy, and autonomous fitness behavior (51), the impact mechanism of social support on autonomous fitness behavior in college students (52), and the influence of new media choices on autonomous fitness behavior of college students (53). In current research, it is not difficult to find that there are relatively few studies focused specifically on autonomous fitness behavior among college students compared to those related to exercise behavior. Moreover, the research on autonomous fitness behavior predominantly originates from China. Additionally, research on autonomous fitness behavior tends to focus on single factors, lacking integrated multi-factor models. The inclusion of research regions also has certain limitations, and the research characteristics involved are summarized in Table 1.

**Table 1:** Characteristics and summary of extracted results from the studies.

Author	Study objectives	Study design	Theoretical framework	Sample characteristics	Sample location	Main results
Sheng (50)	Evaluate the autonomous fitness behavior of college students	Cross-sectional	Knowledge-attitude-practice model; Social exchange theory	*N* = 746 (*M* = 392; *F* = 354)	8 universities in Gansu Province, China	College students have a high level of health awareness and autonomous supportive intentions, but a lower level of awareness of fitness behavior. Most students have not formed autonomous fitness habits and have not achieved good fitness results
Jin et al. (51)	Explore the relationship between exercise disincentives and exercise self-efficacy with autonomous fitness behavior from a cross-sectional and longitudinal perspective	Longitudinal cross-lag	Self-efficacy theory	T1: *N* = 800; T2: *N* = 746 (351M/395F; Mage = 19.76 ± 1.68)	4 universities in Shijiazhuang City, China	Exercise disincentives cannot predict autonomous fitness behavior over time. There is no longitudinal evidence for the mutual influence between exercise disincentives and autonomous fitness behavior
Ni, (53)	Explore the impact of new media choices on autonomous fitness behavior of college students in the post-pandemic era	Cross-sectional	Health belief model; theory of planned behavior; knowledge-attitude-practice model; self-efficacy theory	*N* = 1,227 (*M* = 623; *F* = 604)	5 universities in Zhejiang Province, China	The pandemic has increased college students’ media exposure time and the proportion of audiences focusing on sports content. The choice and use of new media have led to more positive attitudes toward fitness among college students. The effect of new media in promoting autonomous fitness behavior in college students needs to be improved
Li et al. (52)	Explore the impact of perceived social support on autonomous fitness behavior of college students and the mediating role of psychological resilience and exercise self-efficacy	Cross-sectional	Social cognitive theory	*N* = 985 (*M* = 298; *F* = 687; Mage = 19.55)	Universities from five provinces in China: Shandong, Liaoning, Anhui, Henan, Jiangsu, Zhejiang, and Guangdon	Perceived social support can indirectly predict college students’ autonomous fitness behavior through the independent mediating roles of psychological resilience and self-efficacy, as well as the chained mediating effect of both

N*,* sample size; Mage, mean age; M, male; F, female.

## 5 Future directions

### 5.1 Deepening conceptual understanding

Firstly, future research needs to be more precise in defining concepts. Currently, there appears to be a lack of research on the broader concept of autonomous fitness behavior, which could include a set of attributes people possess or achieve related to health or skills (40). The “results” definition part of autonomous fitness behavior seems to lack corresponding research attention. In the narrow sense of fitness behavior, the current autonomous fitness behavior conceptual model proposed by Fang (17) does not include behaviors related to diet, rest, reducing sedentary lifestyle, and other behaviors that maintain a healthy lifestyle. How can the “process” in autonomous fitness behavior promote the “results” in autonomous fitness behavior, i.e., can the narrow definition of autonomous fitness behavior promote the broader concept of autonomous fitness behavior? How are autonomy and agency further transformed in this context? What are the mechanisms of influence? Fitness behavior is a subset of health behavior, which includes a broader range of health-related activities and habits (54, 55). A deeper understanding of the concept can provide a reference and basis for further maintaining and improving the physical health of college students.

Secondly, clearly differentiate autonomous fitness behavior from related concepts such as self-efficacy and understand their differences and connections. This includes a deeper understanding and description of the components, nature, and types of autonomous fitness behavior. Autonomous fitness behavior consists of the sense of self-determination in fitness, the feeling of autonomy support from the environment, and the individual's intentional self-regulation SOC strategies (24). According to Self-Determination Theory (56), the nature of autonomous fitness behavior includes a high degree of personalization, meaning it varies according to individual preferences, goals, capabilities, and resources. At the same time, this behavior is self-directed under environmental influence, meaning that individual planning and behavior are constrained by environmental and situational factors. Additionally, it requires individuals to have adaptability and adjustment capacity, to modify according to changes in life, health status, and fitness outcomes (46). However, the direct differences and connections between types of autonomous fitness behavior in college students (aerobic vs. anaerobic, individual vs. group, indoor vs. outdoor, traditional vs. modern) are still worth further exploration.

### 5.2 How autonomy transitions to persistence

Self-Determination Theory posits that individuals who appear more autonomous tend to persist longer in certain behaviors (57). In the transition from autonomy to persistence, it seems that what is mainly lacking is duration. Studies have shown that becoming a regular exerciser requires at least 6 months of intervention to be effective (58), and 6 months into an exercise program, self-determined motivation may not be sufficient to maintain the behavior. This may be due to the fact that in the process of maintaining physical activity, life stress and barriers are the strongest predictors of persistence (59). At the same time, research indicates that focusing on the emotional outcomes produced by more autonomous regulation during exercise (such as enjoyment) is also a predictor of continued willingness to exercise, exercise habit, and persistence (57, 60, 61). However, there is a lack of deeper longitudinal research in current studies (including the relationship between variables and the temporal validity), and further research is necessary.

In autonomous fitness behavior, the internal sense of self-determination and the perception of autonomy support from the environment (such as enjoying the pleasure brought by exercise, satisfying the desire to improve health or physique, and perceiving institutional, interpersonal, atmospheric, and situational support in the environment) are key antecedents that motivate individuals to start and continue fitness activities (62). After starting to actively engage in fitness, over time, an individual's basic psychological needs or intrinsic motivation are gradually cyclically reinforced following successful experiences (63). Subsequently, individuals can consciously select, optimize, and compensate for fitness goals and methods (64), enabling them to find solutions and maintain fitness behavior when facing challenges such as time management or decreasing motivation. This stage of exercise is considered the phase of autonomous fitness behavior (17).

As time progresses and with repeated practice, fitness behavior gradually transitions from conscious autonomous choices to more automatic habits. In this process, individuals begin to integrate fitness into their daily lives, reducing reliance on willpower. Research indicates that habit formation is a gradual process involving behavior repetition, stability of environmental cues, and the establishment of reward mechanisms (65–67), with automaticity being a necessary condition for habit. The time it takes for an individual to reach a stable state of habit formation ranges from 18 to 254 days, with significant individual variations (68). What individual differences exist in the formation of automatic fitness behavior habits among college students? What adjustments might occur in individuals' attitudes and beliefs during this process? How does emotional response post-exercise influence this transition? Is this process only prevalent in collectivist cultural contexts? And what about its applicability in different cultural, age, or socioeconomic backgrounds? These are all questions worth further exploration in the future.

### 5.3 Technological and interdisciplinary innovations

In recent years, research exploring the integration of modern technologies such as mobile health technology, social media, and virtual reality with modern life has become increasingly common (69–71). Modern society is also becoming more reliant on these innovative technologies. In promoting autonomous fitness behavior among college students, the first consideration seems to be how these technologies adapt to the lifestyles and preferences of college students (72–74). After all, autonomous fitness behavior is just a subset of a healthy lifestyle, and the different needs and preferences of various college students will dictate different categories and themes, such as the use of fitness apps (75). Furthermore, utilizing smartphone applications, wearable devices, virtual reality (VR), and augmented reality (AR) technologies to enhance and monitor the effectiveness and reliability of college students' autonomous fitness behavior has also been a current research focus (76–78). However, what role do these technologies play in the process of behavioral stage changes and the transformation of awareness in fitness behavior stages? What are the mechanisms of impact? These are potential future research directions.

Interdisciplinary research can provide a more comprehensive understanding of sports science (79, 80). It can also help researchers break away from the characteristics of single-discipline research, examine phenomena from different perspectives, and perhaps more effectively utilize knowledge obtained from multiple perspectives to narrow the gap between research and practice (81). The discovery of autonomous fitness behavior itself is the amalgamation of interdisciplinary theories (24). Integrating knowledge from psychology, sociology, medicine, information science, and environmental science, among other fields (82), helps to deepen the understanding and design of interventions that affect college students’ fitness behavior. It explores the dynamic mechanisms of influence and the interconnections between theories to more comprehensively understand and promote autonomous fitness behavior among college students and further improve their physical health status. For example, according to Self-Determination Theory, in addition to autonomy support, support for competence and relatedness is also important for enhancing levels of autonomous motivation (83). A recent educational study identified a classification system of behaviors that support autonomy, competence, and relatedness (84). Researchers can apply these conceptual frameworks to design fitness programs aimed at motivating college students autonomously. This enhanced framework helps understand how various aspects of supporting autonomy affect individuals' motivation to engage independently in fitness activities, facilitating more detailed intervention measures.

Additionally, creating more comprehensive, effective, and sustainable fitness promotion strategies through technological innovation and the integration of interdisciplinary research methods is also a potential future research trend (85–87). At the same time, some models from children and adolescents are also considered applicable to adults (88) and are worth further exploration and verification in the future.

## 6 Conclusion

This narrative review comprehensively analyzes autonomous fitness behavior among college students, highlighting the main conceptual limitations and empirical progress within this field. Despite the integration of Self-Determination Theory, the review reveals ambiguities in accurately measuring autonomous fitness behavior and differentiating it from general exercise behavior, which complicates the implementation of effective interventions. Moreover, although interest in autonomous fitness behaviors is increasing, there is a noticeable lack of comprehensive studies that explore the multifaceted factors influencing these behaviors. Future research should strive to deepen conceptual understanding and further explore the complex dynamics of the transition from autonomy to persistence, employing technological and interdisciplinary methodological perspectives to enhance understanding and promote sustainable fitness habits. This holistic exploration not only deepens our understanding of the complexities of autonomous fitness behavior among college students but also lays the groundwork for developing more effective, targeted health promotion strategies and interventions for this demographic.
